# Robot-assisted resection of ganglion cell neuroma with a diameter of 78 mm: A case report

**DOI:** 10.1097/MD.0000000000039440

**Published:** 2024-09-06

**Authors:** Qipeng Zhang, Hengrui Liang, Jiayang Li, Shouzhuo Li, Enwu Xu

**Affiliations:** a The First School of Clinical Medicine, Southern Medical University, Guangzhou, Guangdong Province, China; b Department of Thoracic Surgery, General Hospital of Southern Theater Command, Guangzhou, Guangdong Province, China; c Department of Thoracic Surgery, The First Affiliated Hospital of Guangzhou Medical University, Guangzhou, China.

**Keywords:** case report, minimally invasive surgery, posterior mediastinal ganglioneuroma, robot-assisted surgery, thoracoscopic surgery

## Abstract

**Rationale:**

Intrathoracic paragangliomas are typically found within the intricate posterior mediastinal region adjacent to the vertebrae, often presenting with substantial volume. Surgical excision of such tumors presents formidable challenges and is conventionally performed via open surgical procedures.

**Patient concerns:**

In this report, we present the case of a 53-year-old female patient who presented with the discovery of a left intrathoracic mass during a routine physical examination approximately 1 month prior. She complained of chest tightness and chest pain.

**Diagnoses:**

She complained of chest tightness and chest pain. Magnetic resonance imaging of the chest and brachial plexus revealed a mass adjacent to the left upper lung hilum, measuring approximately 78 × 63 × 72 mm. The initial suspicion leaned towards a benign lesion. Notably, there was slight compression of the left first thoracic nerve root and mild compression of the middle and lower trunks of the left brachial plexus. Based on the morphological features of the tumor and imaging findings, we suspected its benign nature.

**Interventions:**

We opted for robot-assisted thoracic surgery to resect the mediastinal tumor.

**Outcomes:**

Subsequent postoperative pathology confirmed the diagnosis as a paraganglioma. The patient did not experience any notable complications post-surgery, and a 6-month follow-up revealed no signs of recurrence.

**Lessons:**

The successful application of the robot-assisted thoracic surgery surgical technique underscores its efficacy in minimally invasive resection of sizable intrathoracic tumors situated in the posterior mediastinum.

## 1. Introduction

Paragangliomas, rare benign tumors of neural crest origin, most commonly manifest in the abdomen, thoracic/mediastinal region, or adrenal glands.^[1]^ Early-stage paragangliomas often remain asymptomatic, posing challenges for timely detection. Consequently, they can grow to considerable sizes, with mediastinal tumors generally exhibiting larger dimensions compared to non-mediastinal counterparts.^[2–5]^ Intrathoracic paragangliomas are typically located in the intricate posterior mediastinum, adjacent to vertebral structures.^[6]^ This region houses critical vascular and neural elements, including the aorta, subclavian artery, and recurrent laryngeal nerve. Surgical resection mandates precise separation of the tumor from these vital structures to prevent damage, presenting a significant surgical challenge.^[7]^ Furthermore, the traditional approach for excising larger intrathoracic posterior mediastinal tumors involves open surgery, which may result in heightened postoperative pain, increased complications, and extended hospital stays.^[8]^

Robot-assisted thoracic surgery (RATS) has emerged as a notable advancement in the field of thoracic surgery in recent years.^[9]^ Utilizing robotic systems, RATS provides surgeons with enhanced control over surgical instruments, enabling more precise procedures.^[10]^ This technique combines the benefits of both thoracoscopic surgery and robotic technology, offering an innovative solution for complex surgical cases. In this context, we present a case of RATS for the resection of a substantial left intrathoracic paraganglioma measuring 78 × 63 × 72 mm.

The patient has provided informed consent for the publication of the case. We provide this case report in compliance with the CARE reporting checklist.^[11]^

## 2. Case report

### 2.1. History of present illness

A 53-year-old female patient presented with the complaint of discovering a left intrathoracic mass during a routine physical examination approximately 1 month ago. Additionally, the patient reported occasional chest tightness and pain but did not exhibit symptoms of upper limb numbness.

### 2.2. History of past illness

The patient has no relevant past medical history.

### 2.3. Personal and family history

The patient has no known family history of related diseases.

### 2.4. Physical examination upon admission

Physical examination upon admission revealed no obvious abnormalities.

### 2.5. Imaging examinations

**Figure 1.**
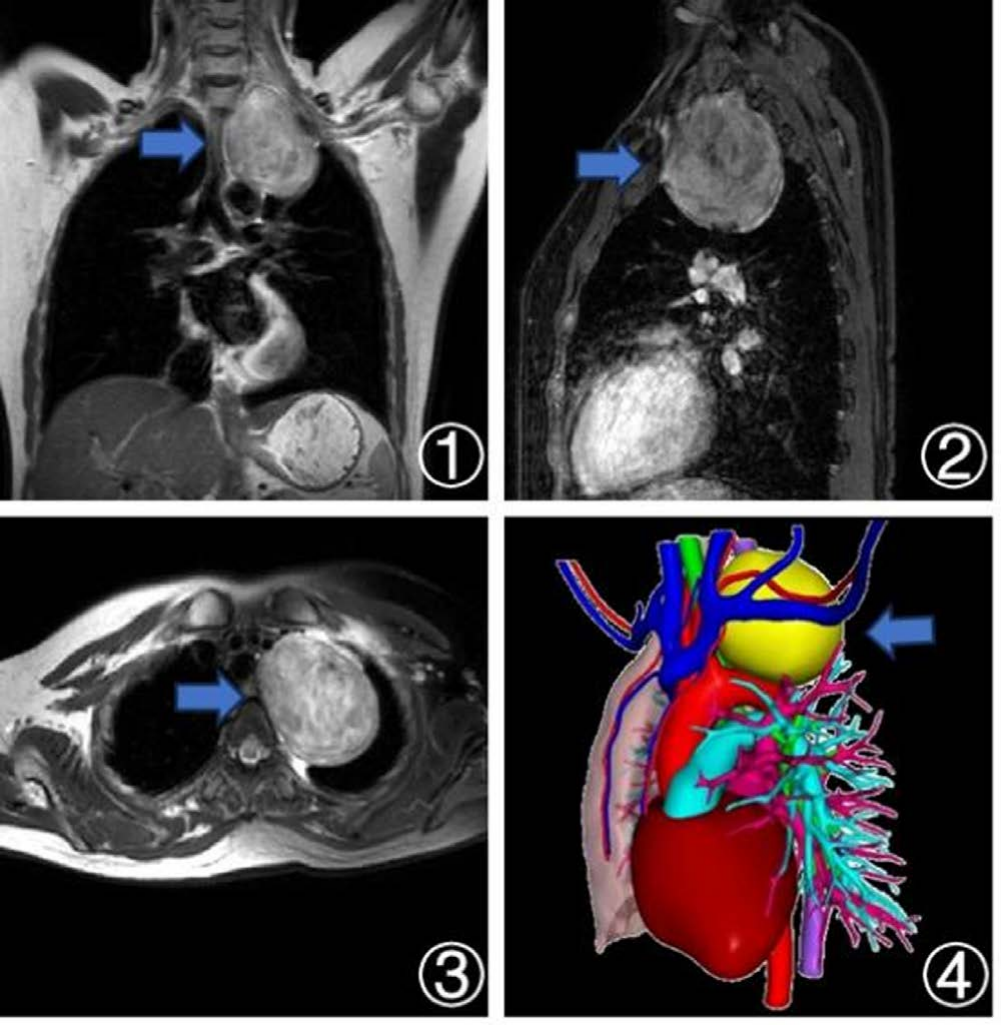
Chest magnetic resonance images and three-dimensional reconstruction model: ① coronal view; ② sagittal view; ③ axial view; ④ three-dimensional reconstruction model of the heart, major blood vessels, and tumor.

A chest computed tomography (CT) scan revealed an oval-shaped consolidation in the upper mediastinal region of the left lung apex, measuring approximately 75 × 56 mm. This mass was situated near the trachea, esophagus, and several blood vessels, leading to compressive effects. A whole-body positron emission tomography computed tomography scan demonstrated uniformly increased metabolic activity within a mass located adjacent to the left upper lobe of the lung and the mediastinum, suggesting an interstitial origin for the tumor. Notably, no highly active enlarged lymph nodes were observed in the hilar regions or mediastinum. Further assessment through magnetic resonance imaging of the chest and brachial plexus revealed a mass adjacent to the left upper lung hilum, measuring approximately 78 × 63 × 72 mm. Based on the observed characteristics, a high likelihood of a benign condition was considered. There was slight compression of the left first thoracic nerve root and mild compression of the middle and lower trunks of the left brachial plexus, as depicted in Figure 1.

### 2.6. Laboratory examinations

Preoperative routine blood analysis indicated mild anemia, while other laboratory tests did not reveal significant abnormalities.

### 2.7. Differential diagnosis

Based on the patient’s clinical presentation and imaging results, the initial diagnosis is considered to be a benign tumor, with a particular inclination towards a neurogenic tumor. However, considering the tumor’s location and imaging characteristics, other possible diagnoses need to be excluded, including neurofibroma, pheochromocytoma, lipoma, malignant fibrous histiocytoma, and metastatic tumors. Neurofibromas are often associated with neurofibromatosis, pheochromocytomas manifest as hypertension and paroxysmal headaches, lipomas are benign tumors of adipose tissue, malignant fibrous histiocytomas are highly malignant soft tissue tumors, and metastatic tumors originate from cancers in other parts of the body. Physical examination tests also play a significant role in differential diagnosis; for instance, cardiac/pulmonary auscultation and blood pressure measurement help identify severe pathologies.^[12]^ Comprehensive physical examination and multimodal imaging (such as CT and magnetic resonance imaging) are crucial steps in confirming the diagnosis and differential diagnosis.

### 2.8. Final diagnosis

The final diagnosis of the presented case was paraganglioma.

### 2.9. Therapeutic

**Figure 2.**
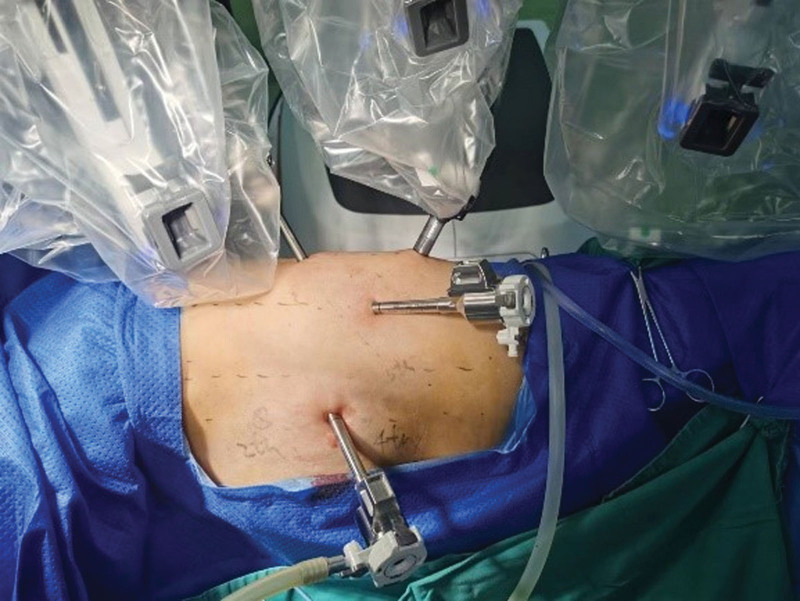
The patient’s position during surgery and the patient’s positioning of the robot.

**Figure 3.**
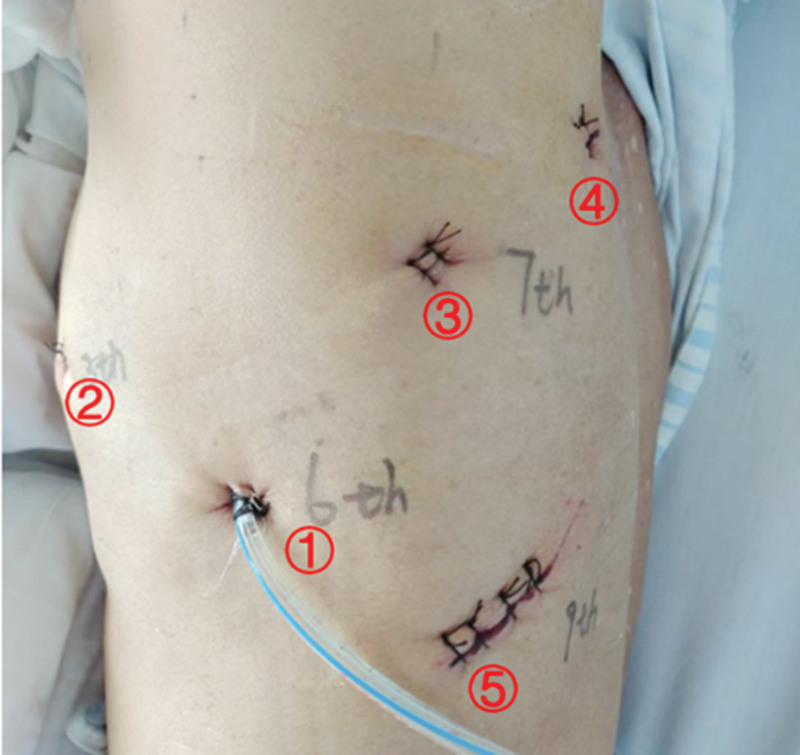
Thoracoscope port, robotic arm port, and auxiliary incision: ① thoracoscope; ② Cardiere forceps; ③ Maryland forceps; ④ Cardiere forceps; ⑤ auxiliary incision.

We opted for RATS to resect the mediastinal tumor. Under the induction of general anesthesia, the patient was positioned in a right lateral decubitus orientation. The Da Vinci Xi robotic system was strategically placed at the patient’s posterior aspect, as illustrated in Figure 2. An 8 mm port was carefully inserted in the left axillary midline at the 6th intercostal space, facilitating the introduction of a thoracoscope into the left thoracic cavity. Additionally, an 8 mm Cardiere port was strategically positioned in the left anterior axillary line at the 5th intercostal space to serve as an access point for instrumentation. Furthermore, an 8 mm Maryland port was meticulously inserted in the left posterior axillary line at the 7th intercostal space. To accommodate the surgical requirements during the operation, a 10 mm auxiliary incision was created in the left infrascapular line at the 9th intercostal space. In addition, a temporary 8 mm Cardiere port was placed at the 7th intercostal space along the left scapular line to facilitate compression and exposure, as depicted in Figure 3.

**Figure 4.**
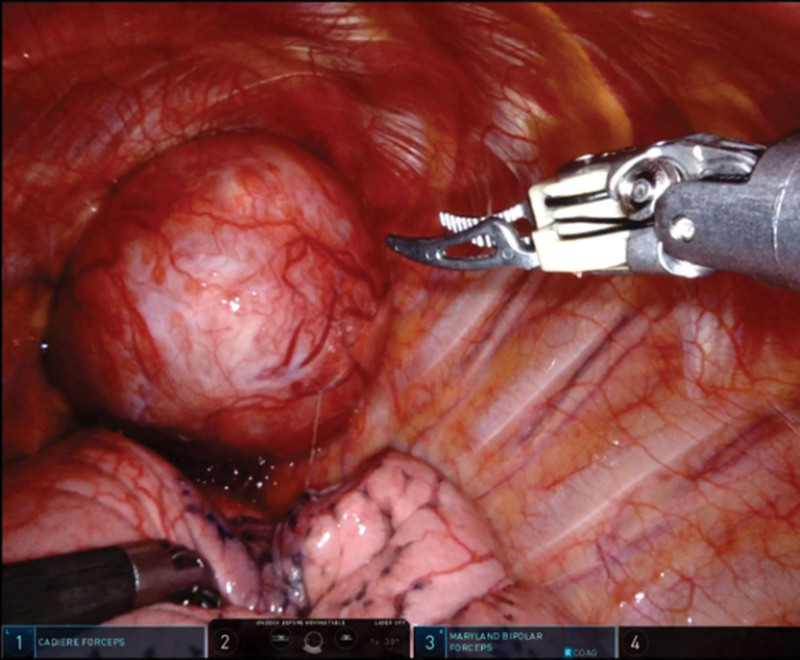
Intraoperative findings of the chest exploration: the tumor is located at the top of the left chest, with a diameter of approximately 100 mm.

**Figure 5.**
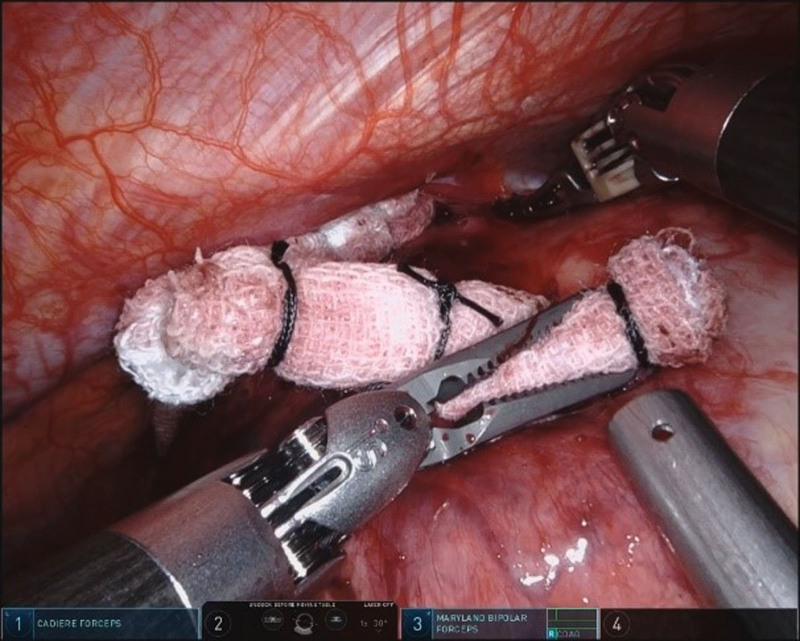
Gauze strips are used to create an adequate operational space and robotic arms operating in the narrow space between the chest wall and the tumor.

**Figure 6.**
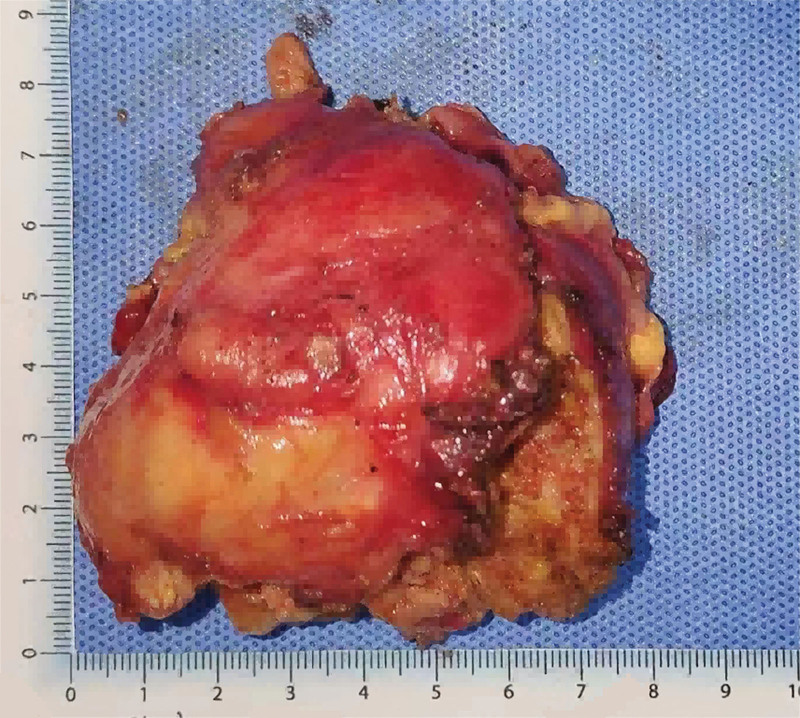
A gross view of the removed tumor.

The surgical procedure unfolded as follows: (I) Intraoperative exploration of the chest revealed minimal adhesions and the presence of fluid. The tumor was situated at the apex of the left lung, with its base adjacent to the aortic arch in the upper anterior mediastinum. Some minor adhesions were noted in the surrounding tissues, and the tumor’s diameter measured approximately 100 mm (see Fig. 4). (II) The procedure commenced by inserting gauze strips into the gap between the anterior base of the tumor and the chest wall. This step was crucial in creating an adequate operational space (see Fig. 5). (III) The surgical team carefully followed the anatomical boundary between the tumor and the chest wall, subsequently making an incision along the visceral pleura surrounding the tumor. During this phase, titanium clips and bipolar electrocautery were utilized to secure and seal off vessels supplying the tumor. (IV) A methodical dissection of the connective tissue around the tumor’s base was carried out to separate the tumor from its attachments. The tumor’s capsule was meticulously dissected free, with the assistance of titanium clips and bipolar electrocautery for the management of tumor-feeding vessels. (V) The tumor’s pedicle was dissected free from its capsule using bipolar electrocautery, and the entire tumor was carefully separated. (VI) Hemostasis was effectively achieved, and the tumor was fragmented using bipolar electrocautery, subsequently being placed in a specimen bag (see Fig. 6). To facilitate tumor extraction, the assistant port at the 9th intercostal space along the left scapular line was extended by 4 cm. A closed chest drain was then positioned in the 6th intercostal space along the left axillary midline.

### 2.10 Follow-up and outcomes

Subsequent to the surgery, the patient received comprehensive postoperative care, encompassing acid suppression, pain management, mucus clearance, and nutritional support. On the third day following the surgical procedure, the chest drainage tube was safely removed, having collected a total drainage volume of 210 mL. The patient was discharged from the hospital 5 days after the surgery, with no noteworthy complications or any incidence of Horner syndrome observed during the hospitalization. The postoperative pathology definitively confirmed the diagnosis of a paraganglioma. The fragmented tumor exhibited dimensions measured 120 × 110 × 30 mm. During a follow-up examination conducted 5 months post-surgery, the patient reported a complete absence of discomfort, including chest tightness or chest pain. A CT scan revealed no abnormalities, such as fluid accumulation or air retention, and most notably, there was no indication of disease recurrence.

## 3. Discussion

Ganglioneuroma is a benign tumor originating from neural crest cells.^[13]^ The etiology of ganglioneuroma is not fully understood, but it is associated with abnormal development of neural crest cells. These tumors can occur in any area containing neural crest cells, but the most common locations are the thorax and abdomen, with 37.5% occurring in the posterior mediastinum.^[2]^ Early-stage mediastinal ganglioneuromas are usually asymptomatic and are often discovered during routine physical examinations. In later stages, their size and location may cause symptoms such as pain or dyspnea due to compression of adjacent structures.^[14]^

Surgical resection is the preferred treatment for mediastinal tumors. Traditionally, these tumors have been removed via standard posterolateral thoracotomy, which is associated with significant postoperative complications such as large incisions, pain, and impaired respiratory muscle function. Thoracoscopic surgery offers benefits like reduced postoperative pain, shorter hospital stays, and improved cosmetic outcomes compared to thoracotomy.^[15]^ However, for larger posterior mediastinal tumors, thoracoscopic surgery is challenging due to limitations in two-dimensional vision and operating angles. The Da Vinci Surgical System allows surgeons to perform complex procedures with enhanced three-dimensional visualization, tremor filtration, and increased instrument mobility,^[16]^ making minimally invasive resection of large posterior mediastinal tumors feasible.

Previous studies have demonstrated the efficacy of RATS in various thoracic surgeries. RATS has been associated with fewer conversions to open procedures, fewer positive margins, shorter hospital stays, and fewer adverse outcomes compared to video-assisted thoracic surgery (VATS) suggesting that RATS may be the preferred technique for minimally invasive resection of mediastinal tumors.^[17]^ Similarly, RATS has shown advantages over VATS in terms of reduced surgical blood loss and shorter postoperative hospital stays for posterior mediastinal neurogenic tumors.^[18]^

This case represents the first instance at our institution of utilizing a robot-assisted surgical procedure to resect a posterior mediastinal tumor exceeding 70 mm in diameter. In this case, the robotic arms’ enhanced range of motion and flexibility allowed for precise maneuvering within the confined surgical field. Techniques such as gauze packing, blunt dissection, and tumor fragmentation facilitated complete tumor dissection and minimally invasive resection without postoperative complications. These advantages highlight the potential of RATS to enhance surgical precision, minimize complications, and improve recovery times.

Our initial experience indicates that robot-assisted resection of large posterior mediastinal tumors is technically feasible and offers excellent perioperative outcomes. This novel technique represents a viable and safe alternative to traditional thoracotomy or thoracoscopic surgery for treating these tumors. RATS is an advanced surgical technique that addresses many limitations of conventional approaches. Its precision reduced trauma, and improved outcomes make it a valuable tool in the clinical management of rare and complex cases.

## 4. Conclusion

In conclusion, RATS is an effective surgical approach for the resection of large posterior mediastinal tumors in the thoracic cavity when the tumor is larger than 70 mm.

## Author contributions

**Conceptualization:** Qipeng Zhang, Hengrui Liang.

**Data curation:** Qipeng Zhang, Shouzhuo Li.

**Formal analysis:** Qipeng Zhang.

**Funding acquisition:** Qipeng Zhang.

**Investigation:** Qipeng Zhang, Shouzhuo Li.

**Methodology:** Qipeng Zhang.

**Validation:** Qipeng Zhang, Jiayang Li.

**Visualization:** Jiayang Li.

**Writing – original draft:** Qipeng Zhang, Hengrui Liang.

**Writing – review & editing:** Enwu Xu.
